# Brazilian best practice guidelines for amblyopia diagnosis and management

**DOI:** 10.5935/0004-2749.2023-0281

**Published:** 2024-11-05

**Authors:** Dayane Cristine Issaho, Júlia Dutra Rossetto, Ian Curi, Roberta Zagui, Luis Carlos Sá, Iara Debert, Aline Brasileiro Pena, Lais Yumi Sakano, Marcia Keiko Uyeno Tabuse, Luisa Moreira Hopker

**Affiliations:** 1 Pediatric Ophthalmology Sector, Hospital de Olhos do Paraná, Curitiba, PR, Brazil; 2 Pediatric Ophthalmology Sector, Universidade Federal do Rio de Janeiro, Rio de Janeiro, RJ, Brazil; 3 Pediatric Ophthalmology Sector, Hospital Federal dos Servidores do Estado do Rio de Janeiro, Rio de Janeiro, RJ, Brazil; 4 Pediatric Ophthalmology Sector, Universidade de São Paulo, São Paulo, SP, Brazil; 5 Centro Brasileiro de Estrabismo, Juiz de Fora, MG, Brazil; 6 Pediatric Ophthalmology Sector, Santa Casa de Misericórdia de São Paulo, São Paulo, SP, Brazil; 7 Pediatric Ophthalmology Sector, Department of Ophthalmology, Hospital São Paulo, Universidade Federal de São Paulo, São Paulo, SP, Brazil

**Keywords:** Amblyopia, Atropine, Contrast sensitivity, Motion perception, Eyeglasses, Visual acuity, Prescriptions

## Abstract

This study aimed to propose a guideline for amblyopia treatment and follow-up. Studies show that amblyopia leads to a series of perceptual deficits, including loss of visual acuity, stereoacuity, and contrast sensitivity. Perceptual changes are also found in the sound eye, such as those involving the types of motion perception. The gold standard of treatment remains the prescription of eyeglasses, when indicated, and patching of the dominant eye. The treatment is mostly effective in patients aged <7 years and must be discontinued gradually, tapering off patching for at least 5 weeks. Atropine may be performed for penalization in hyperopic children whose amblyopic eye has better visual acuity under cycloplegia than the fellow eye. The discovery of significant neural plasticity in the amblyopic brain after the critical period opens possibilities for new treatment modalities even after childhood.

## INTRODUCTION

Amblyopia is a neurodevelopmental disorder leading to reduced visual acuity (VA) in one or both eyes. It is also caused by an abnormal binocular interaction during the critical period of visual development in the first 6-8 years of life, which cannot be attributed to anatomical changes in the visual system^(1)^. Clinically, amblyopia is an interocular difference of two lines or more on a VA test or a VA worse than or equal to 20/30 with the best optical correction^(2)^.

Normal acuity in children may be defined as follows: 20/63 or better at 30-35 months, 20/50 or better at 36-47 months, either 20/40 (or better) or 20/32 (or better) at 48-59 months, and 20/32 or better at 60-72 months of life^(3)^.

Amblyopia is the most common cause of low vision in children in developed countries, affecting 3%-6% of the population^(4)^. In a recent systematic review, the overall worldwide pooled prevalence rate of amblyopia was 1.36%^(4)^, with 1.76%-4.07% in Brazil^(1^, ^4^, ^5^, ^6)^.

The two most common conditions that can disrupt visual development are anisometropia and strabismus, leading to a higher risk of amblyopia. In a population-based study, the relative prevalence of amblyopia was classified as 50% anisometropic, 19% strabismic, 27% mixed strabismic and anisometropic, and 4% from visual deprivation^(7)^.

Amblyopia leads to perceptual deficits, including loss of VA, stereoacuity, and contrast sensitivity, particularly at high spatial frequencies. Perceptual changes can also be found in the sound eye, such as those involving the types of motion perception, reflecting changes in neural responses and functional connectivity in the visual cortex^(8^, ^9^, ^10)^. Additionally, some visuomotor deficits and psychological sequelae may also occur^(10)^.

Currently, guidelines for amblyopia treatment and follow-up have not been established in Brazil. Early diagnoses of ocular changes associated with amblyopia are essential for good visual prognoses because treatment can be started at a stage when the visual neurological pathways are still responsive to stimulation, recovery, and reversal of cortical damage. Therefore, we aimed to propose amblyopia treatment and follow-up guidelines for Brazilian ophthalmologists.

## METHODS

We designed the guidelines based on a review of the literature and the clinical experience of a group comprising members of the Brazilian Center of Strabismus (*Centro Brasileiro de Estrabismo* - CBE) and the Brazilian Society of Pediatric Ophthalmology (*Sociedade Brasileira de Oftalmologia Pediátrica* - SBOP). We conducted a literature review focusing on amblyopia classification, diagnosis, and treatment by searching PubMed up to March 2023. We used the following terms: amblyopia OR lazy eye AND binocular vision OR strabismic OR strabismus OR suppression OR deprivation OR anisometropic OR refractive OR stimulus deprivation-induced OR diagnosis OR treatment OR therapy OR patching OR occlusion OR penalization OR atropine OR pharmacologic penalization OR binocular treatment OR binocular therapy OR dichoptic.

We qualified the studies based on the level of evidence and the method used by Guyatt et al.: Level I was based on two or more high-quality randomized controlled trials (RCTs); studies with a high level of evidence based on the Grading of Recommendations Assessment, Development, and Evaluation (GRADE); or statements from other guidelines with Level A of evidence (experimental or observational studies with higher consistency). Level II was based on a small number of RCTs, more than one controlled but not a randomized study, or more than one RCT of lesser quality; cohort or case-control studies, preferably from more than one research group or center; observations of clear-cut effects in non-controlled studies; studies with a moderate evidence level based on GRADE; or statements from other guidelines with Level B of evidence (experimental or observational studies with lower consistency). Level III was based on expert opinion; clinical experience; descriptive, cohort, or case-control studies of lower quality; studies with low or very low evidence level based on GRADE; or statements from other guidelines with Level C or D of evidence (case reports or specialist opinion-based consensus).

The electronic searches identified 577 titles and abstracts, and 95 scientific papers were selected (30 and 65 were levels I and II of evidence). We excluded level III studies.

All society representatives involved approved the final guideline document, and ethical approval was waived because no human subjects participated.

### Types of amblyopia

Amblyopia is generally classified by its cause (Table 1):

**Table 1:** Mechanisms of amblyopia

Causes	Anomalous binocular interaction	Visual deprivation
Strabismic amblyopia	Yes	No
Anisometropic amblyopia	Yes	Yes
Bilateral high refractive amblyopia	No	Yes
Visual deprivation (unilateral)	Yes	Yes
Visual deprivation (bilateral)	No	Yes

Source: adapted from Von Noorden et al.^(1)^

### Strabismic amblyopia

Due to anomalous binocular interaction, strabismus during the critical period of brain development can lead to amblyopia. Typically, it occurs in constant, not alter-nating deviations, and is always unilateral and caused by active inhibition in the retinocortical pathways of visual input originating from the fovea of the deviating eye^(1)^.

Except in severe cases, the cortical ocular dominance columns usually remain with their normal structure. However, many functional changes occur with the loss of V1 binocular connections^(11^, ^12^, ^13)^. In addition to VA loss, strabismic amblyopia compromises binocular vision and the ability to discriminate disparity and depth of vision due to altered stereoscopic VA (stereopsis), but contrast sensitivity is relatively spared^(14^, ^15)^.

### Refractive amblyopia

#### Anisometropic amblyopia

During the critical period, the eye with the greater refractive error sends less stimulus to the central nervous system in the presence of anisometropia. The blurred image leads to a mild form of deprivation, and the difference in sharpness between the two images represents a form of anomalous binocular interaction, leading to foveal inhibition of the amblyopic eye^(1^, ^16)^. In some cases, the difference between the image sizes of the two eyes (aniseikonia) represents an additional component leading to amblyopia.

Anisometropic amblyopia is more common with the presence of hyperopic anisometropia. In myopic anisometropia, the more ametropic eye can be used in near-vision tasks in some cases, whereas the less myopic eye is used for distance, avoiding the development of more severe amblyopia. The following degrees of anisometropia will likely cause amblyopia: 1.50 D of anisohyperopia, 2.00 D of anisoastigmatism, and 3.00 D of anisomyopia. Thus, amblyopia risk is about twice as high in hyperopic than in myopic anisometropias of comparable refractive imbalance^(17)^.

In pure anisometropic amblyopia, the VA deficits and loss of contrast sensitivity of all spatial frequencies are quite proportional, but binocular vision is relatively spared^(14^, ^15)^. However, it has the best prognosis among all amblyopia types. Patients may recover or dramatically improve their VA with eyeglass prescription alone, even at later ages^(18^, ^19)^. However, anisometropic amblyopia is often associated with microtropia and other strabismus types, leading to a mixed mechanism^(1)^ that increases amblyopia severity. For the same degree of anisometropia, the VA for strabismus is on average, which is 2.5 times worse than for a non-strabismic case with similar anisometropia^(17)^.

### Bilateral high-refractive amblyopia (isometropia)

Bilateral high-refractive errors cause bilateral symmetric retinal blurred images with no competitive images between the two eyes. Thus, if the refractive error is not corrected, a form of continuous visual deprivation occurs, leading to bilateral amblyopia^(1)^. This type of amblyopia occurs more commonly in high-hyperopic (wherein the magnitude of the refractive error exceeds the accommodative tolerance) or in high-astigmatic patients (meridional amblyopia). However, it rarely occurs in high-myopic children because they can use their near vision to adequately stimulate the visual areas despite their blurry distant vision^(20)^.

### Deprivation amblyopia

Complete or incomplete obstruction of the visual axis causes deprivation amblyopia, preventing the light stimulus from reaching the retina. The lack of visual stimulus during the critical period leads to anatomical and functional changes in the visual pathways. These changes are more intense with earlier occurrences and the longer the deprivation is^(21^, ^22^, ^23^, ^24)^. The damage is located mainly in V1, with changes in their ocular dominance columns^(21)^, and morphological changes in the lateral geniculate body^(23)^.

The major causes of deprivation amblyopia are infantile cataracts, blepharoptosis, and corneal opacities. Unilateral deprivation amblyopia is more severe than bilateral deprivation amblyopia because of the superim-posed mechanisms of binocular anomalous interaction and visual deprivation^(21^, ^22^, ^23)^.

Some structural diseases, such as optic nerve hypoplasia, retinopathy of prematurity, and macular scars from congenital toxoplasmosis, may have a treatable deprivation amblyopia component (or strabismic and/or anisometropic amblyopia components) in addition to the visual loss that could be attributed to structural damage^(25)^. Therefore, amblyopia treatment should be attempted even in eyes with structural damage.

Deprivation amblyopia is the most severe form of amblyopia, and the critical period for its treatment is extremely short^(26)^, with frustrating results. The final VA depends directly on the cause of the deprivation and the promptness of treatment.

### Diagnosis

Screening for amblyopia and its risk factors in very young children provides the best opportunity for effective treatment. The preferred method of screening for amblyopia in childhood is direct measurement of the best-corrected monocular VA using optotype-based charts, which should be performed as early as possible^(27)^. Children aged between 3 and 5 years are considered the most effective age group for large-scale screening for amblyopia^(28)^.

Children younger than 5 years old may not cooperate with subjective VA testing. Although fixation preference testing may be an imperfect method for diagnosis^(29^, ^30)^, it is currently the most widely accepted for deciding which preverbal children need treatment. The 10-diopter fixation test can be very useful for children with small-angle tropias and those without strabismus. Vertical deviation is induced by placing a 10-diopter vertical prism over one eye. Once the eyes are dissociated, fixation preference is evaluated and used to predict the presence of amblyopia.

**Figure 1:**
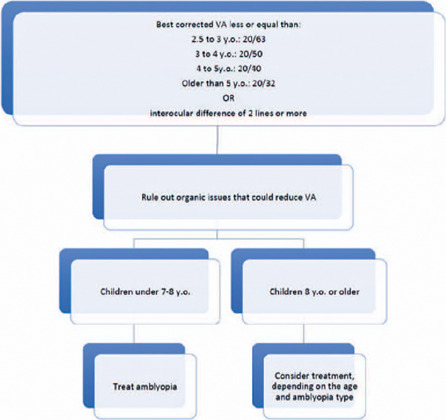
Diagnosis and treatment decision fuxogram.

The Teller acuity cards are another behavioral assessment to quantify VA in younger children^(27)^ Furthermore, the American Academy of Pediatrics recommends instrument-based vision screening of preverbal children^(31)^ to detect amblyopia risk factors and select patients to be examined by an ophthalmologist^(32)^. Additionally, electrophysiology tests, such as visual evoked potential, can also be used to measure VA in preverbal children and children with developmental delays^(33)^. Figure 1 shows the diagnosis flowchart.

### Treatment

The gold standard treatment of amblyopia involves the use of eyeglasses (when necessary) and dominant eye occlusion to force the brain to use inputs from the eye with less VA, enabling the cortex to overcome suppression, recover connections, and improve the development of visual functions of the amblyopic eye. Some alternatives to occlusion include penalizing the dominant eye with 1% atropine eye drops, filtering lenses, optical blurring with glasses or contact lenses, and, more recently, using binocular stimuli (dichoptic treatment).

Over the past 25 years, the Pediatric Eye Disease Investigator Group (PEDIG)^(34)^ and the Monitored Occlusion Treatment of Amblyopia Study (MOTAS)^(35)^ have conducted RCTs to address key issues in amblyopia treatment and proposed optimal treatment protocols (Level 1 of evidence).

PEDIG has published >20 amblyopia treatment studies that have evaluated the treatment of amblyopia in children aged 3-17 years, with the following results:

1. Optical correction alone can improve amblyopia in almost one-third of patients^(18^, ^36)^.

2. Occlusion can effectively treat amblyopia^(37)^.

3. The ideal number of occlusion hours depends on the severity of amblyopia.

3.1. For children with moderate amblyopia (worse than 20/30 to 20/100), 2-h occlusion daily is ideal.

Two groups were randomized to use either 2 or 6-h of occlusion daily. Although the 6-h occlusion group achieved faster results, both groups achieved similar final VA at the end of 4 months of treatment^(38)^.

3.2. For children with severe amblyopia (worse than 20/100 to 20/400), 6-h occlusion daily is ideal.

Two groups were randomized to use either 6-h of occlusion daily or full-time occlusion. Both groups obtained favorable results in VA at the end of the treatment period^(39)^. However, a higher number of occlusion hours was associated with worse adherence to treatment^(40)^.

However, these studies did not consider the different types of amblyopia and other variables, such as the prescribed hours of patching and the real duration of patching. Therefore, the recommended number of hours should not be interpreted as the new occlusion prescription guidelines. Occlusive treatment should be customized for each patient, based on the onset of amblyopia and its different etiologies, as well as adherence and treatment outcome^(18)^.

4. Atropine penalization is as effective as occlusion. Atropine penalization was prescribed in seven important trials. For 3-7-year-old participants with moderate amblyopia (0.3 to 0.6 LogMAR, 20/40 to 20/80 Snellen equivalent), weekend atropine and daily atropine dosages improved vision similarly (PEDIG 2004)^(41)^. Although VA improved faster in the occlusion group, both groups achieved equivalent improvements in VA at the end of 6 months of treatment, which were maintained over a long follow-up period (up to 15 years). In addition to daily atropine, once weekly atropine improved VA, with better adherence to treatment. Thus, atropine penalization should be performed in hyperopic children whose amblyopic eye has better VA under cycloplegia than the fellow eye^(42)^.

5. Amblyopia treatment is most effective in patients younger than 7 years. However, the VA of children up to 13 years old significantly improved with occlusion, although with a slower rate of response to treatment, incomplete recovery, and the need for a greater amount of occlusion^(43)^.

6. The recurrence rate is high after amblyopia treatment, and treatment tapering is highly recommended.

Recurrence after treatment occurs in approximately 25% of the patients, which was similar for occlusion and atropine. rate was four times higher in children who did not undergo occlusion tapering for at least 5 weeks after amblyopia resolution. Other factors associated with high rates of relapse were better VA at the end of treatment, a greater number of lines of improvement, and previous history of relapse^(44)^.

7. Performing occlusion associated with near-vision activities is highly controversial. Some studies showed better results in children who performed these tasks^(45^,^46)^, whereas other studies reported that performing common near-vision activities did not improve the VA outcome when treating anisometropic, strabismic, or combined amblyopia with 2- of daily patching^(47)^.

8. Treatment of amblyopia with levodopa for residual amblyopia was not statistically significant^(48)^.

9. Binocular treatments using dichoptic strategies might be as effective as occlusion therapy.

Dichoptic treatments were studied in pilot projects, which aimed to improve VA and binocularity in patients even after the critical period of visual plasticity with interesting results. However, these treatments were not superior to classic occlusion in RCTs in the final improvement of VA or stereopsis. New methods and technologies are being developed and studied to improve treatment adherence and perhaps obtain better results in global visual functions^(49^, ^50^, ^51)^.

### Treating amblyopia outside the critical period

Although the young brain has greater plasticity, adult brains can still learn and recover after injury. Therefore, plasticity is present at the synaptic, cellular, and cortical levels of representation in adults^(52^, ^53^, ^54)^.

Various intrinsic and extrinsic forms of plasticity stimulation have been used to facilitate amblyopia treatment after the critical period of development. Intrinsic stimulation can be achieved through environmental or behavioral manipulation of the neurotransmitter systems regulating synaptic plasticity: exercises and improvement of the visual environment, prolonged exposure to the dark, caloric restriction, and new or challenging visual tasks^(53^, ^55^, ^56^, ^57^, ^58^, ^59^, ^60)^. Moreover, extrinsic stimulation involves exogenous manipulation of the endogenous neuromodulatory system, such as the use of levodopa.

**Figure 2:**
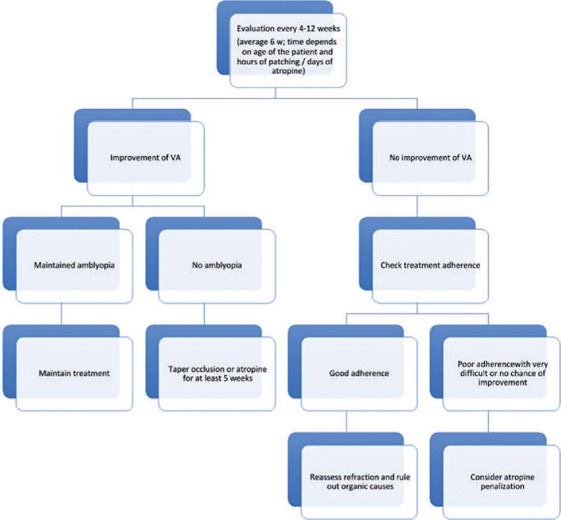
Treatment fowchart.

However, a randomized, placebo-controlled clinical trial conducted by PEDIG showed that VA improvement with levodopa was did not show a statistically significant difference compared with placebo in patients subjected to recurrent amblyopia treatment. Moreover, the treated group did not maintain VA improvement during follow-up after discontinuing the medication^(48^, ^61)^. Another option would be using substances altering gene expression to remove molecular “obstacles” to cortical plasticity^(58^, ^62^, ^63^, ^64^, ^65)^. Figure 2 shows the treatment flowchart.

IO VA*: interocular visual acuity.

### Amblyopia as a binocular disease

Typically, amblyopia affects VA more commonly in one eye; thus, it has been considered a monocular disease. Therefore, the main treatment consists of dominant eye occlusion to improve the monocular function of the amblyopic eye. Nonetheless, several studies show that visual loss in amblyopia extends beyond monocular impairment and adversely affects higher-order visual functions, such as binocular vision, fixation stability, reading speed, and visuomotor activities, due to abnormal interocular interactions^(15^, ^66^, ^67^, ^68^, ^69)^. However, these deficiencies are not corrected with monocular treatment and persist even after VA is recovered after occlusion.

Thus, amblyopia is intrinsically a binocular problem, and visual suppression should be addressed during amblyopia treatment rather than waiting for binocular vision to improve after the improvement of monocular VA with occlusive therapy. Thus, new binocular treatments have been proposed. Therefore, Hess, Mansouri, and Thompson recommended a treatment based on strengthening the binocular matching of the images by gradually reducing suppression^(70^, ^71)^.

The strategy used was to present each eye with images with different contrasts (maximum and minimum contrasts in the image presented to the amblyopic and dominant eyes, respectively) to combat suppression and enable the normalization of binocular interactions to recover binocular vision. With this binocular approach, individuals with strabismic amblyopia can combine the information from both eyes^(8)^. These authors proposed dichoptic treatment, a new type of treatment for amblyopia. This concept has been applied in both active and passive forms of treatment for amblyopia. Passive training modalities include watching movies under dichotic viewing conditions^(72^, ^73)^. Additionally, active training uses video games that require binocular matching to complete the game objective^(74^, ^75^, ^76^, ^77^, ^78^, ^79^, ^80^, ^81^, ^82^, ^83^, ^84)^.

PEDIG has conducted large-scale RCTs involving 5-13-year-old patients and compared the effects of binocular dichoptic stimulation with occlusion for 2-h daily for 16 weeks. These studies have shown low adherence to the prescribed game and video regimen, and improvement in VA and stereopsis with these strategies was not greater compared with 2-h of daily occlusion. Further research was recommended using other more attractive strategies and games to improve treatment adherence, such as adventure and action games, shooting games, virtual reality, and three-dimensional game platforms^(7^, ^53^, ^85^, ^86^, ^87^, ^88^, ^89)^. Because treatment offers children the choice of unlimited streamed visual content to keep them engaged, with continued support from the monitoring center, treatment adherence will likely remain high even outside the rigor of a clinical study^(50)^.

Although the dichoptic treatment did not show superior improvement over VA occlusion and stereopsis, VA improvement was similar in all protocols and in patients’ performance during games, indicating better binocular interaction and less suppression. Studies show that binocular movie treatment at-home improved amblyopic eye BCVA after 2 weeks (similar to patching), with additional improvement up to 6 weeks. Additionally, repeated binocular visual experience with contrast-rebalanced binocular movies provides an additional treatment option for amblyopia^(90)^.

Therefore, improvement of other visual functions altered in amblyopia should be evaluated. This depends directly on normal binocular interaction, such as Vernier acuity, contrast sensitivity at different complexity levels, global movement tasks, fixation stability, and quality of life, to assess individuals’ subjective perception of changes in their vision^(50^, ^85^, ^91^, ^92^, ^93)^.

A global and more careful study of individuals with amblyopia could help us better define, understand, and classify this disorder, aiding physicians in determining a more personalized and effective treatment and explaining the high variability of the patients’ responses to the treatment, with failures in some cases^(49^, ^52)^.

### Follow-up

Children treated for amblyopia must be closely monitored to allow sufficient time for possible changes in therapeutic strategies. Follow-up is performed every 6 weeks on average (range, 4-12 weeks). The characteristics of amblyopia, as well as the child’s particularities, determine the safest intervals between visits. The most relevant variables include the type and severity of amblyopia, VA, modality and intensity of the proposed treatment, history of previous treatments and adverse effects, age, psychosocial characteristics of the child and family, and treatment adherence^(37^, ^94)^.

The treatment strategy should be maintained with progressive improvement but should be replaced by another modality or intensified if without further improvement. If no satisfactory result is obtained, reassessing the refractive status and checking adherence to treatment are recommended. When decreased VA occurs in the dominant eye, consider the diagnosis of reverse amblyopia to discontinue treatment^(94^, ^95)^.

**Figure 3:**
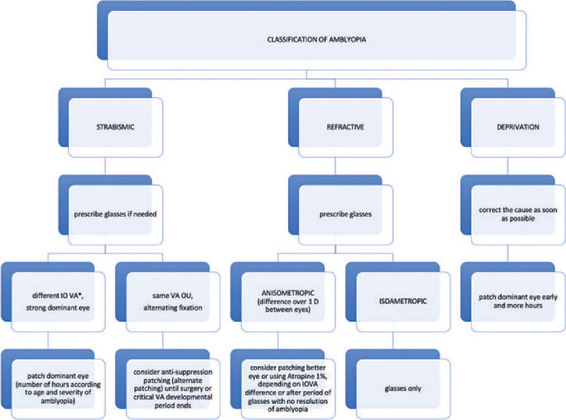
Follow-up flowchart.

Treatment should be continued until VA is equalized in both eyes or stabilized at a plateau. Once maximal VA is achieved, treatment should be tapered off slowly (for at least 5 weeks) before being stopped. Follow-up visits are still necessary even with discontinued treatment to assess any regression. Recurrence occurs in approximately a quarter of successfully treated children within the first year after treatment discontinuation, and it may occur in patients who discontinue the occlusion, as well as in those who discontinue penalization with atropine^(95^, ^96)^. Figure 3 shows the follow-up flowchart.

## DISCUSSION

Amblyopia is the most common cause of monocular visual loss in children and a considerable public health issue^(1^, ^4^, ^6)^. Due to impaired stereoacuity, motor skills, motion perception, and fixation stability, individuals with amblyopia may have difficulties when performing daily activities with quality-of-life implications extending beyond visual problems in children and adults, impacting reading speed, multiple-choice test answer (Scantron) completion time, family life, social interactions, economic status, and emotional and mental health^(97)^.

Early diagnosis to enable treatment during the visual development period is recommended and highly effective, significantly improving the quality of vision and of life^(97)^. However, treatment is not uniform worldwide, with no standardized guidelines for amblyopia management. Thus, variations in practice patterns exist among and within countries due to political, societal, and economic factors.

Over the past decades, groups such as PEDIG^(34)^ and MOTAS^(35)^ have conducted RCTs to address key issues in amblyopia treatment and define optimal treatment protocols. Undoubtedly, traditional amblyopia therapies can be efficacious with timely intervention and good compliance, but the occurrence of residual amblyopia is common, and these therapies mainly use monocular approaches.

Therefore, traditional interventions may not provide a completely comprehensive illustration of visuomotor behavior and the full impact of amblyopia on daily quality of life. Additionally, they may not assess the full treatment effect of different therapies.

Recent research on amblyopia provides new concepts and better understanding regarding this common vision-threatening clinical condition. Thus, we currently understand that amblyopia is a binocular condition. The dichoptic treatment showed similar improvements in VA and performance of patients during games, indicating binocular interaction and less suppression with either treatment. One of the most important factors affecting the success of amblyopia treatment is compliance. Previously, binocular therapy was an effective solution for those with poor treatment adherence, but several studies showed that compliance was better in patients treated with patching. New methods and technologies are being developed and studied to improve treatment adherence and perhaps obtain better results in global visual functions^(49^, ^50)^.

Also, neural plasticity is considerable in the amblyopic brain beyond the critical period, potentially stimulating amblyopia treatment at later ages.

Improvement of other visual functions altering amblyopia should be evaluated, depending on normal binocular interaction.

Finally, other factors, such as cost and availability, should be considered when selecting the most appropriate amblyopia therapy, particularly when considering different socioeconomic and geographical contexts.

After a thorough scientific review of amblyopia, this consensus document was written. Thus, the CBE and SBOP aimed to establish guidelines for diagnosing, treating, and monitoring amblyopia, considering the clinical and demographic aspects of this condition in Brazil.

Recent research on amblyopia has introduced new concepts and provided a better understanding of amblyopia. The gold standard treatment for amblyopia remains the use of spectacles, when indicated, as well as patching of the dominant eye. After achieving the expected VA, treatment must be discontinued gradually, tapering off patching for at least 5 weeks. Atropine for penalization may be performed in hyperopic children whose amblyopic eye has better VA under cycloplegia compared with the fellow eye. Amblyopia treatment is the most effective in patients younger than 7 years old. The discovery of significant neural plasticity in the amblyopic brain after the critical period has potentially provided possibilities of new treatment modalities, even during adolescence and adulthood.
